# *Cyp26b1* Expression in Murine Sertoli Cells Is Required to Maintain Male Germ Cells in an Undifferentiated State during Embryogenesis

**DOI:** 10.1371/journal.pone.0007501

**Published:** 2009-10-19

**Authors:** Hui Li, Glenn MacLean, Don Cameron, Margaret Clagett-Dame, Martin Petkovich

**Affiliations:** 1 Departments of Pathology and Molecular Medicine, Queen's University, Kingston, Ontario, Canada; 2 Department of Biochemistry, Queen's University, Kingston, Ontario, Canada; 3 Division of Cancer Biology and Genetics, Cancer Research Institute, Queen's University, Kingston, Ontario, Canada; 4 Department of Biochemistry, University of Wisconsin-Madison, Madison, Wisconsin, United States of America; 5 Pharmaceutical Science Division, University of Wisconsin-Madison, Madison, Wisconsin, United States of America; Ecole Normale Supérieure de Lyon, France

## Abstract

In mammals, germ cells within the developing gonad follow a sexually dimorphic pathway. Germ cells in the murine ovary enter meiotic prophase during embryogenesis, whereas germ cells in the embryonic testis arrest in G0 of mitotic cell cycle and do not enter meiosis until after birth. In mice, retinoic acid (RA) signaling has been implicated in controlling entry into meiosis in germ cells, as meiosis in male embryonic germ cells is blocked by the activity of a RA-catabolizing enzyme, CYP26B1. However, the mechanisms regulating mitotic arrest in male germ cells are not well understood. *Cyp26b1* expression in the testes begins in somatic cells at embryonic day (E) 11.5, prior to mitotic arrest, and persists throughout fetal development. Here, we show that Sertoli cell-specific loss of CYP26B1 activity between E15.5 and E16.5, several days after germ cell sex determination, causes male germ cells to exit from G0, re-enter the mitotic cell cycle and initiate meiotic prophase. These results suggest that male germ cells retain the developmental potential to differentiate in meiosis until at least at E15.5. CYP26B1 in Sertoli cells acts as a masculinizing factor to arrest male germ cells in the G0 phase of the cell cycle and prevents them from entering meiosis, and thus is essential for the maintenance of the undifferentiated state of male germ cells during embryonic development.

## Introduction

Retinoic acid (RA) is a vitamin A derived signaling molecule that regulates cell proliferation, migration, and differentiation during embryonic development and adult homeostasis. RA-mediated signaling is controlled in embryonic tissues through coordinated regulation of RA synthesis and catabolism. Synthesis is catalyzed by a family of retinaldehyde dehydrogenases (ALDH1A1, ALDH1A2 and ALDH1A3) that irreversibly oxidize retinal to form RA, while catabolism is facilitated by a family of cytochrome P450 enzymes (CYP26A1, CYP26B1 and CYP26C1), which convert RA to more polar, inactive metabolites [1], [2]. Therefore, the distribution and activity of these enzymes define where RA signaling will occur. Gene targeting studies have demonstrated that changing the endogenous distribution of RA can have severe consequences for the developing embryo. *Aldh1a2^−/−^* embryos die around embryonic day (E) 10.5 and display phenotypes resembling severe maternal vitamin A deficiency, while *Cyp26a1* and *Cyp26b1* null embryos exhibit numerous malformations reminiscent of RA teratogenicity [3]–[8].

Recent genetic studies suggest that RA plays a role in the development of embryonic germ cells [9]–[11]. In mice, primordial germ cells (PGCs) migrate from the proximal epiblast and reach the developing gonad between E10 and 11. PGCs continue to divide mitotically for a few days and then germ cells in males and females follow a sexually dimorphic pathway. In males, in response to a hypothesized unidentified masculinizing factor, germ cells arrest in G0 phase in the mitotic cycle [12], [13]. Cells re-enter the cell cycle a few days after birth, and the first meiotic spermatocytes are seen at postnatal day (P) 10 [14]. In contrast, female germ cells continue through the cell cycle to enter prophase of the first meiotic division, and progress through leptotene, zygotene and pachytene, arresting in diplotene just before birth [15]. In the embryonic male gonad, *Cyp26b1* transcripts are detected in somatic cells as early as E11.5 and persist throughout development [16]. *Cyp26b1* expression is detected in peritubular myoepithelial cells in the postnatal testis, while *Cyp26b1* expression is absent in developing and adult ovaries [17]. *Aldh1a2* expression in the gonad initiates at E10.5 in the mesonephros and is maintained until at least E13.5. Postnatally, *Aldh1a2* transcripts are detected at P1, and expression increases significantly until P20 when protein is detected in pachytene spermatocytes, and later in the adult expression is restricted to round spermatids [17]. Therefore, in the male gonad, the expression of *Aldh1a2* and *Cyp26b1* acts as a ‘source’ and ‘sink’ of RA, thus defining when and where RA-mediated signaling will occur. Increasing evidence has suggested that entry of germ cells into the prophase of the first meiotic division requires RA and its responsive gene *Stimulated by retinoic acid gene 8* (*Stra8*) [9]–[11], [18]. Our analysis of *Cyp26b1^−/−^* embryos revealed increased levels of RA in the embryonic testes. RA exposure at this stage causes male germ cells to prematurely enter meiosis, followed by apoptosis [9]. Furthermore, expression of three meiotic genes, *Stra8*, synaptonemal complex protein 3 (*Scp3*) and dosage suppressor of mck1 homolog (*Dmc1*) are elevated in E13.5 testes of *Cyp26b1* null embryos [10], indicating that CYP26B1 activity prevents entry of male germ cells into meiosis. CYP26B1 activity may also regulate mitotic arrest in male germ cells, as it has been shown *in vitro* that RA promotes mitosis in cultured gonads [19].

The continued expression of *Cyp26b1* in embryonic testes beyond E13.5 suggests a role for CYP26B1 at later stages in regulating male germ cell development. We have generated a Sertoli cell-specific knockout mouse line of *Cyp26b1* by crossing floxed *Cyp26b1* (*Cyp26b1^fl/fl^*) animals with mice expressing Cre recombinase under the control of the anti-Müllerian hormone (*Amh*) promoter. As the *Amh*-*Cre* transgene is expressed only in Sertoli cells from E15 onwards [20], this conditional knockout mouse line of *Cyp26b1* allows us to define the role of CYP26B1 in male germ cells after E15, when they are already committed to the male developmental pathway.

## Materials and Methods

### Generation and genotyping of *Cyp26b1^SC−/SC−^* mice

Mice in which exons 3–6 of the *Cyp26b1* locus are flanked by loxP sites have been previously described [9]. These animals (*Cyp26b1^fl/fl^*) were crossed with mice expressing Cre recombinase under the control of the *Amh* promoter (*Amh-Cre*) [20] and resultant offspring were intercrossed to excise both alleles of *Cyp26b1* in Sertoli cells (*Cyp26b1^SC−/SC−^*). Using DNA extracted from tail clips, mice were genotyped by PCR for both *Cyp26b1* and Cre recombinase. Presence of a floxed *Cyp26b1* allele was detected using the primers P1 (5′-CAGTAGATGTTTGAGTGACACAGCC) and P2 (5′-GAGGAAGTGTCAGGAGAAGTGG) which flank the 5′ loxP site. These primers will amplify a product of 223 bp from a wild-type allele and 284 bp from a targeted (L3) allele. In order to detect a null allele, DNA was extracted from testes, and PCR was performed with the primers P1, P2 and P3 (5′-GGGCCACCAAGGAAGATGCTGAGG), as P1 and P3 will amplify a product of 364 bp from an excised allele. Cre recombinase was detected using the primers 5′-AGGGATCGCCAGGCGTTTTC and 5′-GTTTTCTTTTCGGATCCGCC. All PCR was performed using Taq Polymerase (Sigma-Aldrich, St. Louis, MO), and included 1.5 mM MgCl_2_, 0.2 mM dNTPs and 0.2 mM of appropriate primers. Each PCR consisted of 30 cycles of 94°C for 20 sec, 58°C for 45 sec and 72°C for 50 sec.

For embryo collection, mating females were checked in the morning for vaginal plugs, if present, this was denoted as E0.5. Embryos were collected in ice-cold PBS, and fetuses were decapitated prior to tissue collection. All animal experimentation was reviewed and approved by the Queen's University Animal Care and Use Committee.

### RNA extraction and reverse transcription–polymerase chain reaction (RT-PCR)

Total RNA was isolated from freshly dissected gonads with Trizol reagent (Invitrogen, Carlsbad, CA) according to the manufacturer's recommendations. Total RNA (1 µg) was reverse transcribed with random hexamers (0.2 µg) using 30 units avian myeloblastosis virus reverse transcriptase (Promega Madison, WI) in a total reaction volume of 25 µL. PCR was conducted by using 1 µL of cDNA as template in a total volume of 20 µL. The following primer sets were used: Stra8: 5′-CTGTTGGACCAGATGCTGAA-3′ and 5′-GCAACAGAGTGGAGGAGGAG-3′; and mouse vasa homolog (Mvh): 5′-TGGCAGAGCGATTTCTTTTT-3′ and 5′-CGCTGTATTCAACGTGTGCT-3′. All PCR was performed using Advantage 2 Polymerase Mix (Clontech, Mountain View, CA). Each PCR consisted of 35 cycles of 95°C for 30 sec and 68°C for 1 min.

### Tissue collection and histology

Freshly dissected testes from *Cyp26b1^SC−/SC−^* and appropriate control mice were fixed for 1 h in Bouin's solution, washed in 70% ethanol and paraffin embedded. Sections (5 µm) were cut, dewaxed, and stained with hematoxylin and eosin as previously described [9]. At least four testes from mice of each genotype were examined at each developmental stage.

### Immunofluorescence

For immunofluorescence (IF) experiments, sections were blocked in 5% serum (matched to the species of the secondary antibody) in PBS for 30 min at room temperature and then incubated with primary antibodies overnight at 4°C prior to detection with secondary antibodies. Primary antibodies used for IF were rabbit anti-mouse vasa homolog (MVH, 1∶2000, provided by T. Noce), mouse anti-Ki67 (1∶400, BD Bioscience), rabbit anti-SCP3 antiserum (1∶500, provided by C. Heyting) [21], rat anti-TRA98 (1∶1000, provided by H. Tanaka and Y. Nishimune), and rabbit anti-3ß-hydroxysteroid dehydrogenase (3ßHSD) (1∶5000; provided by A. Payne). Secondary antibodies used were goat anti-rabbit Alexa 488, goat anti-rat Alexa 594 and goat anti-mouse Alexa 594 (1∶500, Molecular Probes). Sections were counterstained with nuclear stain 4′6-diamindino-2-phenylindole (DAPI). Images were obtained using a Nikon Eclipse TE 2000-U microscope. Negative controls, lacking the primary antisera, were included in each experiment.

In order to determine the mitotic index of germ cells, the total number of Ki67-stained or unstained germ cells (MVH-labeled cells) in the entire cross section was counted under a microscope. At least five cross sections were analyzed for each developmental stage.

## Results

### Sertoli cell specific excision of *Cyp26b1*

**Figure 1:**
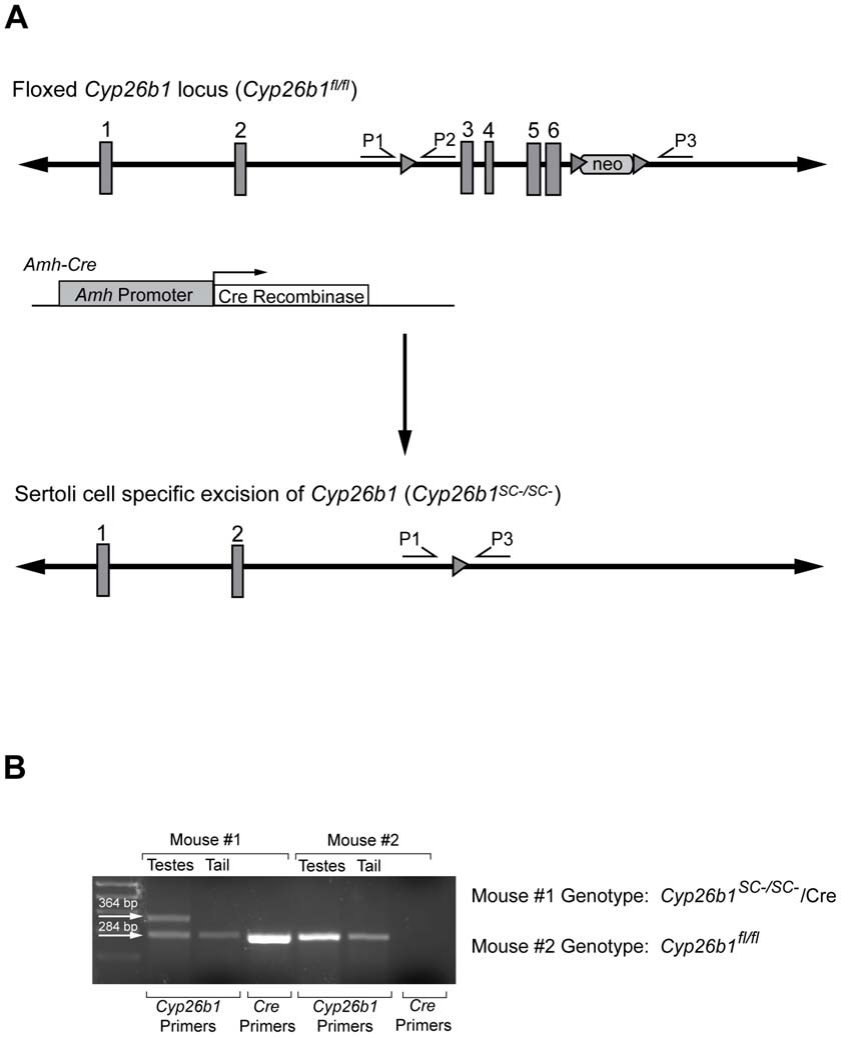
Generation of Sertoli cell-specific *Cyp26b1* knockout mice (*Cyp26b1^SC−/SC−^*). (A) Floxed *Cyp26b1* locus showing position of primers (P1, P2, P3) used for genotyping. LoxP sites are indicated by triangles, and the exons of *Cyp26b1* are numbered. In Sertoli cells, exons 3–6 will be excised *(Cyp26b1^SC−/SC−^)*, thus allowing for PCR amplification of a 364 bp product using P1 and P3. (B) PCR genotyping using P1, P2 and P3 showing detection of an excised allele (364 bp) only in the testes of a *Cyp26b1^fl/fl^* mouse also expressing Cre (mouse #1).

Mice in which exons 3–6 of *Cyp26b1* (*Cyp26b1^fl/fl^*) are flanked by loxP sites (Figure 1A) were generated as previously described [9]. These animals were crossed with *Amh-Cre* mice, which express Cre recombinase specifically in Sertoli cells from E15 onwards [20]. Appropriate crosses were performed to generate mice in which both alleles of *Cyp26b1* were excised in Sertoli cells, known hereafter as *Cyp26b1^SC−/SC−^* mice (Figure 1A). In order to confirm specificity of Cre activity in *Cyp26b1^SC−/SC−^* mice, DNA was extracted from tails and testes of *Cyp26b1^SC−/SC−^* animals as well as wild-type littermates harboring the *Amh-Cre* transgene (*Cyp26b1^SC+/SC+^*). Genotyping was performed using appropriate primers, and excision of *Cyp26b1* was detected only in the testes of *Cyp26b1^SC−/SC−^* animals (Figure 1B, 364 bp band). It should be noted in the testes of *Cyp26b1^SC−/SC−^* mice, a band corresponding to a floxed *Cyp26b1* allele was still detected by PCR (Figure 1B, 284 bp band). This may indicate that there was not 100% excision of the *Cyp26b1^fl/fl^* locus, or may reflect amplification of DNA extracted from non-Sertoli cells in the testes.

**Figure 2:**
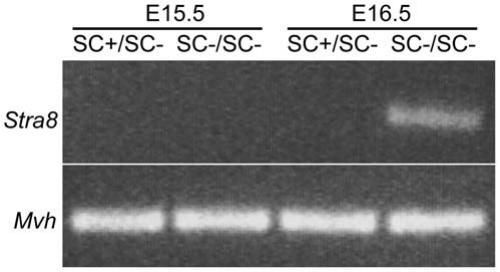
Stra8 expression is elevated in the absence of CYP26B1. Reverse transcription- PCR was performed with RNA collected from E15.5 and E16.5 *Cyp26b1^SC+/SC−^* and *Cyp26b1^SC−/SC−^* testes. *Stra8* is only detected in RNA from E16.5 *Cyp26b1^SC−/SC−^* testes. *Mvh* expression was analyzed as a positive control for RNA integrity.

We next sought to determine the timing of loss of CYP26B1 activity in *Cyp26b1^SC−/SC−^* mice by examining the expression of *Stra8*, a RA-responsive gene. *Stra8* expression is absent in wild type embryonic testes. In *Cyp26b1* ^−/−^ embryonic testes, *Stra8* expression is induced [10]. RT-PCR analysis showed no *Stra8* expression in either *Cyp26b1^SC+/SC−^* or *Cyp26b1^SC−/SC−^* testes at E15.5 (Figure 2), indicating that RA levels remained unchanged at E15.5. At E16.5, *Stra8* mRNA was detected in *Cyp26b1^SC−/SC−^* testes but not in *Cyp26b1^SC+/SC−^* testes (Figure 2). *Mvh* was used as a marker of germ cells, and observed to be expressed in all analyzed samples.

### Testis degeneration in *Cyp26b1^SC−/SC−^* mice

**Figure 3:**
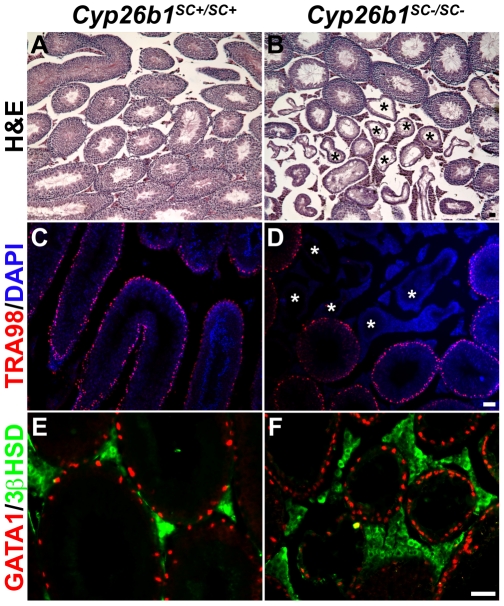
Loss of germ cells in adult *Cyp26b1^SC−/SC−^* animals. 3-month-old *Cyp26b1^SC+/SC+^* and *Cyp26b1^SC−/SC−^* testes stained with hematoxylin and eosin (A and B) or antibodies to TRA98 (a germ cell marker, red in C and D), GATA-1 (a Sertoli cell marker, red in E and F) and 3βHSD (a Leydig cell marker, green in E and F). Note the decrease in the seminiferous tubule size in testes from *Cyp26b1^SC−/SC−^* mice (F) compared to *Cyp26b1^SC+/SC+^* mice (E). Seminiferous tubules devoid of germ cells are indicated by asterisks. Bars, 20 µm.

*Cyp26b1^SC−/SC−^* mice were healthy, viable and did not show any abnormalities of external genitalia. However, they had smaller testes than littermates having at least one wild-type *Cyp26b1* allele. Morphological evaluation of testes from 3-month-old *Cyp26b1^SC−/SC−^* mice revealed an incompletely penetrant phenotype of abnormal seminiferous tubules, with some devoid of all germ cells (Figure 3A, B, asterisks) while other tubules contained cells from all stages of spermatogenesis. IF with the germ cell specific antibody TRA98, confirmed that germ cells were depleted in some seminiferous tubules of *Cyp26b1^SC−/SC−^* animals (Figure 3C, D). Other cell types were examined using antibodies to GATA1 (Sertoli cell specific marker) and 3βHSD (Leydig cell specific marker). It was also noted that in *Cyp26b1^SC−/SC−^* samples, the diameter of seminiferous tubules lacking germ cells was reduced, and Leydig cell hyperplasia was evident outside these tubules (Figure 3F). Such abnormalities were not observed in *Cyp26b1^SC+/SC+^* or *Cyp26b1^fl/fl^* control testes (Figure 3, A, C, E, data not shown). In contrast, numbers of Sertoli cells were unchanged between mutant (Figure 3F) and control animals (Figure 3E).

### Loss of gonocytes in *Cyp26b1^SC−/SC−^* neonatal testes

The observed depletion of spermatogenic cells could be due to either i) the disruption of spermatogenesis, or ii) the loss of gonocytes, which are precursor cells of spermatogonia. In order to distinguish between these two possibilities, IF was performed on sections from *Cyp26b1^SC−/SC−^* testes at P0 using the germ cell-specific antibody TRA98.

**Figure 4:**
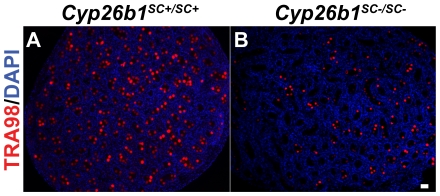
Neonatal loss of germ cells in the absence of CYP26B1. Postnatal day 0 (P0) testes stained for TRA98 show a loss of germ cells in *Cyp26b1^SC−/SC−^* mice. Bar, 20 µm.

Overall, *Cyp26b1^SC−/SC−^* testes contained fewer germ cells in comparison with *Cyp26b1^SC+/SC+^* testes (Figure 4). In contrast to *Cyp26b1^SC+/SC+^* testes where germ cells were present in all seminiferous tubules, (Figure 4A), some seminiferous tubules were devoid of germ cells in *Cyp26b1^SC−/SC−^* testes (Figure 4B), indicating that there is loss of germ cells by P0.

### Meiotic germ cells are detected in E16.5 *Cyp26b1^SC−/SC−^* testes

**Figure 5:**
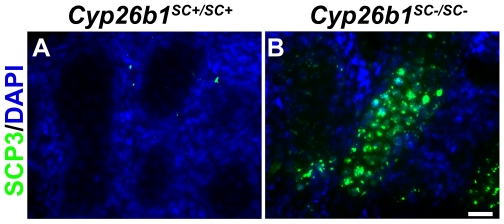
*Cyp26b1^SC−/SC−^* male germ cells enter meiosis prematurely. Sections of testes from *Cyp26b1^SC+/SC+^* (A) and *Cyp26b1^SC−/SC−^* (B) littermates at E16.5 stained for the meiotic marker SCP3 (green). Sections were counterstained with DAPI. Bar, 20 µm.

Previously, we observed that loss of CYP26B1 function by E11.5 resulted in the inappropriate initiation of meiotic prophase in testes. As *Stra8* expression is not induced at E15.5 in *Cyp26b1^SC−/SC−^* mice, testes were collected and sectioned from E15.5 and E16.5 *Cyp26b1^SC−/SC−^* and control fetuses. Sections were stained for the synaptonemal complex protein SCP3, a marker indicative of meiosis, which is upregulated in *Cyp26b1^−/−^* testes [9], [10]. At E15.5, no SCP3-stained cells were seen in either *Cyp26b1^SC+/SC+^* or *Cyp26b1^SC−/SC−^* testes (data not shown). At E16.5, in contrast to *Cyp26b1^SC+/SC+^* testes in which no SCP3-stained cells were observed, SCP3-positive cells were detected in a subset of tubules in *Cyp26b1^SC−/SC−^* testes (Figure 5).

### Germ cells exit from G0 and re-enter mitosis in E16.5 *Cyp26b1^SC−/SC−^* testes

**Figure 6:**
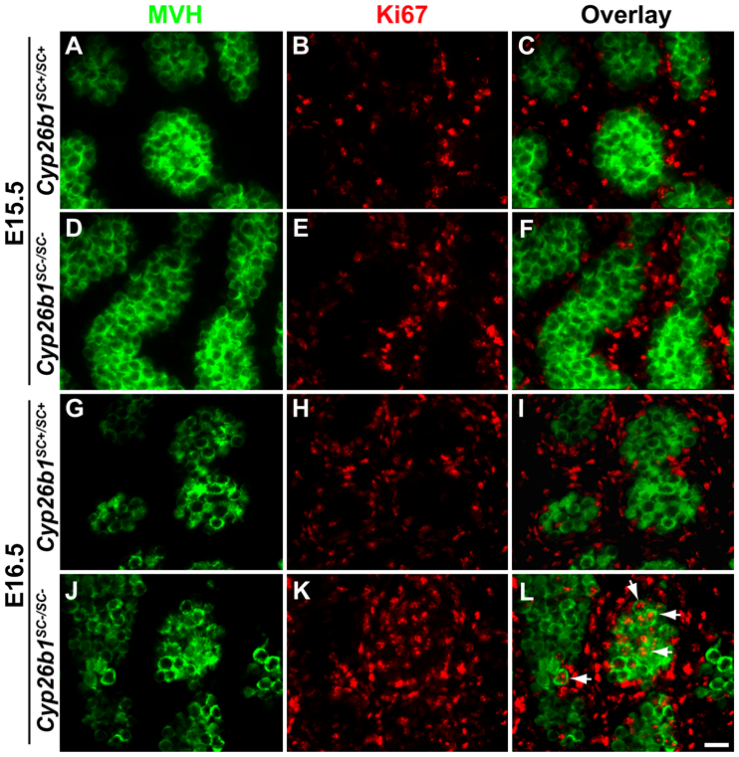
Re-entry into mitotic cell cycle in embryonic *Cyp26b1^SC−/SC−^* male germ cells. Sections of testes from *Cyp26b1^SC+/SC+^* (A, B, C, G, H, I) and *Cyp26b1^SC−/SC−^* (D, E, F, J, K, L) littermates at E15.5 (A–F) and E16.5 (G–L) stained for the mitotic marker Ki67 (B, E, H, K, red) and the germ cell marker MVH (A, D, G, J, green). Overlays of images show Ki67 expressing germ cells are observed only in *Cyp26b1^SC−/SC−^* fetuses (F, arrowheads). Bar, 20 µm.

In order to determine if CYP26B1 is required for maintaining mitotic quiescence in male germ cells, E15.5 and E16.5 testes were sectioned and IF was performed with antibody to Ki67 (Figure 6B, E, H, K), which detects all phases of the cell cycle except for G0 and is a common marker of mitotic cells. Samples were also labeled with the germ cell-specific antibody, MVH (Figure 6A, D, G, J). At E15.5, *Cyp26b1^SC+/SC+^* and *Cyp26b1^SC−/SC−^*testes contained very few Ki67-positive germ cells (<1%), while somatic cells stained with Ki67 (Figure 6). At E16.5, virtually no Ki67 expressing germ cells were detected in *Cyp26b1^SC+/SC+^* testes (Figure 6I), whereas 22% of the germ cells were Ki67-positive in the testes of *Cyp26b1^SC−/SC−^* mice (Figure 6L, arrowheads). There was no significant difference in the overall number of Ki67-stained somatic cells between *Cyp26b1^SC−/SC−^* and control mice.

## Discussion

We previously reported that genetic deletion of *Cyp26b1* by E11.5, prior to the germ cell sex determination, leads to increased RA levels in the embryonic testes resulting in premature meiotic initiation and apoptosis in male germ cells [9]. In the present study, we have specifically knocked out *Cyp26b1* in Sertoli cells from E15.5 onwards. We observed induction of *Stra8* expression and initiation of meiosis in germ cells as early as E16.5, and a loss of germ cells by P0, resulting in abnormal seminiferous tubules devoid of germ cells in adult testes. Furthermore, it was observed that some male germ cells at E16.5 exit from G0 and enter the cell cycle, a process that normally does not occur until a few days after birth. These results suggest that male germ cells retain the ability to commit to male or female development until at least E15.5. CYP26B1 in Sertoli cells during embryonic development keeps male germ cells undifferentiated by arresting them in mitotic quiescence and preventing them from meiotic entry until after birth, when mitosis resumes and meiosis initiates as required for spermatogenesis.

It has been proposed that all germ cells enter meiosis cell autonomously unless prevented from doing so by an unidentified meiosis-preventing substance (MPS) in the embryonic testis [12]. Germ cells enter meiotic prophase during embryogenesis not only in ovaries, but also in non-gonadal regions where germ cells escape the inhibitory influence of MPS. When male germ cells from E10.5 or E11.5 gonads are cultured with disaggregated embryonic lung cells, they enter meiotic prophase, but germ cells from older male embryos (E12.5 and E13.5) do not enter meiosis [12]. Based on these observations, it has been proposed that the decision in germ cells to commit to a male or female pathway is made between E12.5 and 13.5 [12]. Previously, it was shown that in *Cyp26b1^−/−^* fetuses, male germs cells enter meiosis at E13.5 [9]. In the *Cyp26b1^SC−/SC−^* model that we have described, CYP26B1 activity is not lost until between E15.5 and E16.5, when male germ cells arrest in G0 and are normally already committed to develop along a male pathway. However, in *Cyp26b1^SC−/SC−^* fetuses, meiotic germ cells are detected at E16.5, suggesting that male germ cells at E15.5 still retain the potential to initiate meiosis and that the commitment of these cells to the male developmental pathway is reversible. These observations demonstrate that CYP26B1 functions as a MPS to prevent male germ cells from entering meiosis in various stages of development, and without its inhibitory influence, male germ cells enter meiosis even though they are already committed to the male developmental pathway. Altogether, these observations suggest that entry into meiosis is the result of an inductive program subject to RA signaling, rather than a cell-intrinsic event.

Importantly, we find that *Cyp26b1^SC−/SC−^* male germ cells exit from G0 and re-enter the mitotic cell cycle. In mice, germ cells within the developing gonad undergo sexually dimorphic cell cycles between E12.5 and E15.5. This developmental switch is dependent on the sex of the somatic cells, rather than the sex chromosome constitution of the germ cells [12]. It has been proposed that a masculinizing factor(s) produced by somatic cells direct male germ cells to arrest in G0 of the cell cycle instead of entering the meiosis as female germ cells do at the same time [12]. The finding that *Cyp26b1^SC−/SC−^* male germ cells exit from G0 and re-enter the mitotic cell cycle is significant, as it shows that CYP26B1 activity in Sertoli cells not only prevents meiosis in germ cells, but is also required for maintaining mitotic arrest in the developing testes, suggesting that CYP26B1 (due to its RA-catabolizing activity) is a candidate masculinizing factor. Furthermore, our findings demonstrate that male germ cells at E15.5 and older retain the ability to continue through the mitotic cell cycle and enter meiotic prophase in response to RA signaling. Thus, CYP26B1 activity in Sertoli cells keeps male germ cells undifferentiated during embryonic development. It has been reported that RA promotes the proliferation of both cultured PGCs [22] and embryonic male germ cells , but has no effects on the somatic cell proliferation [23]. We have provided *in vivo* evidence that male germ cells must remain in an RA-depleted state to enter mitotic arrest. In the mouse testes, germ cells arrest in G0 of the cell cycle between E12.5–E15.5, which coincides with the expression of *Cyp26b1* in the male gonad. Germ cells remain in mitotic quiescence until shortly after birth when mitosis is resumed as required for spermatogenesis [24]. This timing corresponds with testicular expression of *Aldh1a2*, as ALDH1A2 is first detected in the developing testis at birth, and levels increase postnatally [17]. Collectively, these results suggest that RA signaling may also play a role in maintaining the mitotic activity in both PGCs and undifferentiated spermatogonia in postnatal testis.

**Figure 7:**
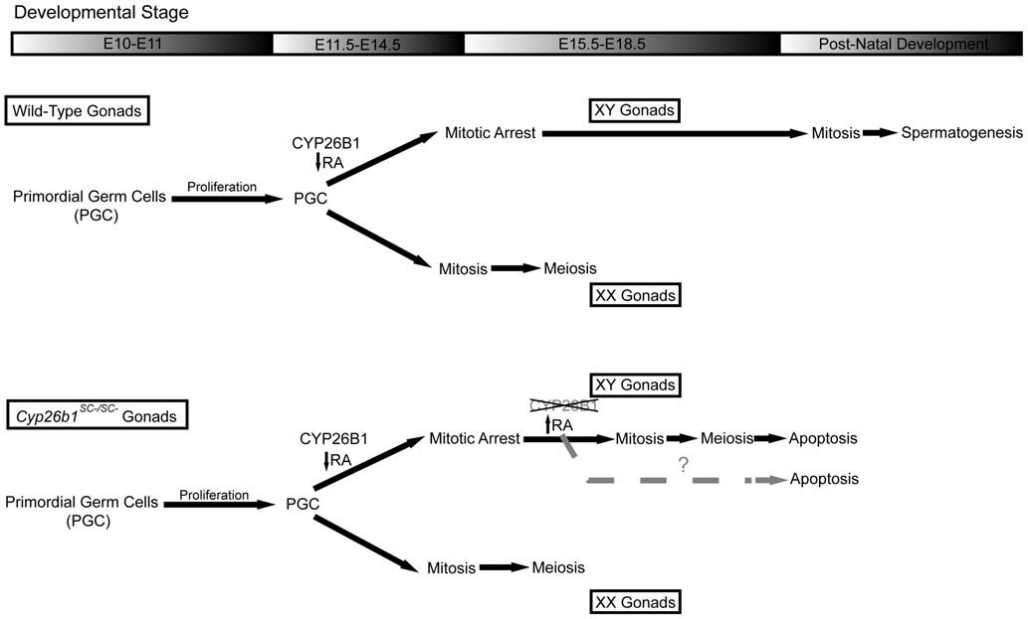
Proposed model for the role of CYP26B1 in maintaining male germ cells in an undifferentiated state during embryogenesis. In wild-type gonads, germ cells exhibit sex-specific divergence during embryogenesis as male germ cells enter mitotic arrest, while female germ cells enter mitosis followed by meiosis. However, in *Cyp26b1^SC−/SC−^* fetuses, Cyp26b1 activity is inactivated after E15.5, thus elevating levels of retinoic acid within the testes. As a result, male germ cells exit from G0 to re-enter the cell cycle and initiate meiotic prophase, which subsequently culminates in loss of male germ cells.

In the embryonic testes, the ALDHs and CYP26B1 act as a source and sink for RA. It has been proposed that RA is synthesized in the mesonephros by ALDH1A enzymes, and diffuses into adjacent cells until reaching Sertoli cells where it is catabolized by CYP26B1. This process is essential for germ cell maturation, as an RA-depleted environment is required for the entry of male germ cells into mitotic arrest and thus prevents germ cells from entry into meiotic prophase. CYP26B1 in Sertoli cells directs male germ cells to develop along a male pathway (mitotic quiescence) and maintains the undifferentiated state of the male germ cells from E11.5 onward. However, the absence of CYP26B1 in Sertoli cells enables endogenous levels of RA to rise in the testes at a time when they should be low or absent. Subsequently, germ cells exit the G0 stage to re-enter the cell cycle and initiate meiotic prophase (Figure 7). At this point, we hypothesize that a factor is released from Sertoli cells that either directly, or indirectly initiates apoptosis of germ cells. The identity of such a factor(s), and mechanisms underlying initiation of apoptosis are currently under investigation.
